# The Influence of Fiber Tension During Filament Winding on the Modal Parameters of Composite Pressure Vessels

**DOI:** 10.3390/polym17152071

**Published:** 2025-07-29

**Authors:** Aleksander Kmiecik, Maciej Panek

**Affiliations:** Department of Mechanics, Materials and Biomedical Engineering, Faculty of Mechanical Engineering, Wrocław University of Science and Technology, 27 Wybrzeże Stanisława Wyspiańskiego st., 50-370 Wrocław, Poland; aleksander.andrzej.kmiecik@gmail.com

**Keywords:** composite pressure vessel, modal analysis, fiber tension, prestress

## Abstract

The aim of this paper is the investigation of changes in modal parameters of composite pressure vessel structures with different prestress states realized by varying fiber tension. Two series of vessels was manufactured and examined with different wound tensions, the first—3 N and second—80 N, respectively. Other technological factors, such as the type and weight of carbon fiber used, as well as liner type, were kept constant. The vessels were examined with internal pressure equal to atmospheric and without pressure fittings. The modal tests were performed on storage tanks suspended on an elastic cord in the horizontal orientation to prevent the structure from being disturbed by vibrations. The examinations were focused only on the cylindrical part of the vessels. Based on modal analysis, parameters such as natural frequencies, dampings and modal shapes were determined. Research results indicate clear changes in natural frequencies and damping coefficients between the two investigated prestress states. It is interesting that natural frequencies for bending modes are higher in the case of structures with high fiber tension, while in the case of other vibration forms, the natural frequencies have smaller values in comparison with the first series.

## 1. Introduction

High-pressure vessels are crucial for storing and transporting gases and liquids, particularly in green energy solutions, such as hydrogen storage, and in the aerospace, rocket, and spacecraft industries.

Regarding the actual state of the art, the pressure vessels used in industry can be divided into five groups. Starting with type I—fully metallic, very cheap and widely used to type V, which relies only on carbon fiber composite. In both cases, the material of container fabrication ensures structural integrity as well as hermetic medium storage [1]. Vessels type II–IV, known as composite overwrapped pressure vessels (COPVs), utilise a liner for gas containment (in type IV made of polymeric material and the earlier two, a metallic one) and a composite to withstand part or all the structural load (in type II, fiber wound only on the cylindrical part) [2].

In COPVs production, two variants of filament winding methods are used—dry and wet. In the first method, dry fibers are wound onto the liner and subsequently infused with resin, while in the second method, fibers are pulled through a resin bath just before being wound.

The filament winding process during the manufacturing of composite pressure vessels has a crucial impact on the properties of the final product. The mechanical properties of the vessel are determined by the structure as a whole as well as the features of its elements. The properties of the composite shell are affected by the matrix and fiber type, and the process parameters such as winding angle, number of layers, and curing cycle. Another one is the tensile force of the fiber during high-tech manufacturing. The elevated fiber tension (prestress) leads to an increase in nominal pressure, by inducing compressive stress on the steel liner, as well as higher mechanical properties of composite laminate and an improvement in gravimetric efficiency [3,4,5,6,7,8] also the reduction in thermal stresses [9].

Vibration and noise are one of the key factors affecting the device’s durability and comfort perception of the mechanical systems. Therefore, the modal frequencies and damping are considered important parameters related to the study of the dynamic the behavior of fiber-reinforced composite structures.

The research concerning the vibrations of composites of various compositions and shapes is extensive. Most of them concentrate on composite material damping capabilities [10,11,12,13], while some others focus on composite structures [14]. The research results corresponding to vibrations of composites involving the modal frequencies, damping, and shapes are accessible [11,15,16], yet studies regarding composite vessels [17] are scarce, especially those focused on high-pressure containers.

The influence of composition and the prestress changes on frequencies and damping (e.g., damping decrement or damping loss factor) was investigated [13,18,19,20,21,22,23]. There are also research results available concerning the exploitation of the modal analysis of cylinders, [24,25], especially numerical ones. However, most papers focus on changes in the quantitative fraction of the fiber, rather than the matrix, and their influence on modal parameters. Lack of experimental investigations concerning the modal analysis of composite pressure vessels.

The present work focuses on determining the changes in modal parameters of pressure vessels caused by increased fiber tension, which causes prestress and changes in the volume fraction of the composite matrix rather than the fiber.

## 2. Materials and Methods

In order to investigate the influence of stresses occurring during the operation of composite tanks on their modal parameters, 6 tanks divided into 2 groups were selected for testing, each of which had a different value of the programmed prestress. To induce different internal stress states, in the first group, the lowest accessible technological tension of 3 N was used, while in the second group, the highest possible fiber tension of 80 N was chosen; in composite vessel production, normal fiber tension is 10 N.

Afterwards, each vessel was tested using experimental modal analysis, using impulse excitation to determine the natural frequencies, their corresponding vibration modes, and the damping coefficient.

In the case of winding vessels with increased fiber tension, prestress is introduced into the composite, which changes the volume of the matrix while the amount of fiber remains constant [3,5]. It can be considered only as an effect, but also, as in our case, a possibility.

### 2.1. Description of the Analyzed Composite Vessels

Two groups of composite containers were prepared, which were made using a steel liner, carbon fiber, and epoxy resin, known as type III. All tanks were prepared using the same materials and winding process parameters with one exception, two different fiber tension forces were used.

**Figure 1:**
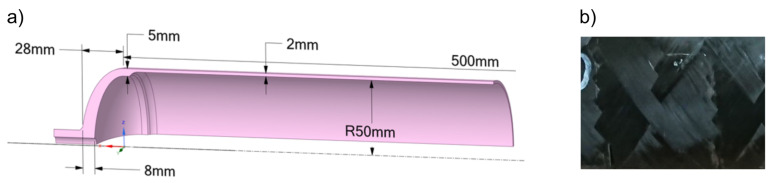
A metallic liner (**a**) and the mosaic pattern (**b**) details.

It was decided to choose steel liners taking into account aspects such as: the increased strength of the steel liner compared with the polymer liner—which was particularly important in the dome area, which due to the fiber winding angle was not optimally covered with a composite coating (composite shell); high modulus of elasticity of steel compared with the stiffness of polymer—prevents liner buckling during the winding process; elimination of composite relaxation during the winding process—which occurs when using polymer liners, caused by the rheological properties of polymers. The liner consist of cylinder 500 mm long with 100 mm inner diameter and two domes with bosses, all of them made of steel and connected using welding method. The liner dimensions can be found in Figure 1.

The tanks were made using a Bolenz & Schafer (Biedenkopf-Eckelshausen, Germany) winder with the wet winding method. The tension of the carbon fiber was controlled by a pneumatic set of spools from EHA Composite Machinery (Steffenberg, Germany). Crucial Winding parameters were as follows: the angle of winding was equal to 54° (the mosaic pattern is shown in Figure 1b), in both winding processes, the entire cylindrical surface was covered by the same length of carbon fiber, the weight of a single composite vessel depended on the amount of resin—EPIKOTE Resin L 20 (Westlake Epoxy B.V., Rotterdam, The Netherlands) used during the winding process, carbon fiber—Torayca 700S 24 k (TORAYCA, Abidos, France) was used with a diameter equal to 7 µm—based on the technical documentation. To obtain a stable temperature during the process of resin hardening, a heat chamber was used. The curing temperature was equal to 30 °C, and vessels were continuously rotated. A detailed description of the analyzed samples series is presented in Table 1.

**Figure 2:**
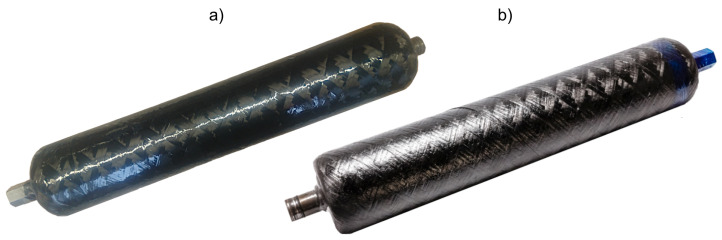
Overview of the vessels used in this study: (**a**) group *L*—winded with low fiber tension; (**b**) group *L*—winded with high fiber tension.

One of the problems revealed during the winding process was the breakage of the fibers at increased tension (80 N). This phenomenon occurred on the winding machine in some fiber guide elements. The effect was also visible on the surface of the tanks, which had a more uneven surface compared with the *L* series tanks, wound with a fiber tension force of 3 N. The *H* series was characterized by a matte surface with a less visible mosaic pattern, which was caused by the mixture of chaotically arranged broken fibers with the resin (compare Figure 2a,b).

### 2.2. Modal Test Setup

**Figure 3:**
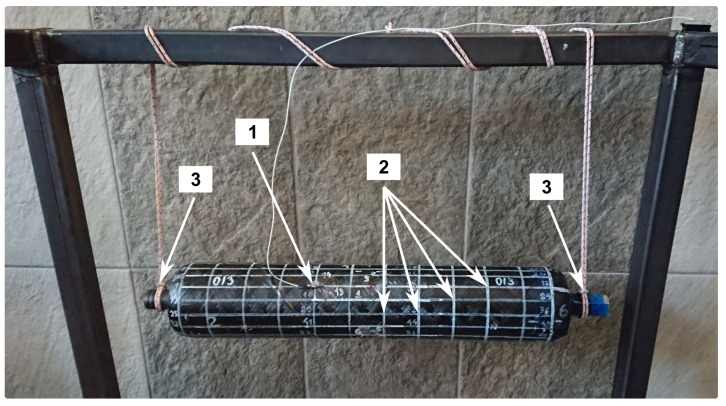
Research stand for modal analysis: 1—accelerometer; 2—measurement points grid; 3—support points.

The examined tanks were horizontally supported, suspended at the elastic cord attached to the metallic bosses at the sides of the vessels (Figure 3). The vessels were tested empty and without pressure fittings—inside pressure was equal to atmospheric. Such an experiment setup ensures being closer to free boundary conditions and allows for the separation of the examined structure from external factors.

For two tanks, one from each group, measurements were performed for the full grid of points, which means that measurement points were located on the entire surface of the cylindrical part of the tank. In that case, the 216 points were distributed in 18 sections arranged along a cylinder and evenly spaced through the circumference. Each section consists of 12 points spaced, as shown in Figure 3. For other vessels, the authors decided not to perform the test on the whole cylinder but only in one randomly chosen section located along a long line of the cylinder. In this case, the measurement net consists of 12 points, located in the chosen section spaced as in the previous case.

In order to determine modal parameters and avoid the change in mass distribution during the experiment, the roving hammer method was chosen. The impulse excitation was applied by a modal hammer (PCB 086C03) with a metal tip, three times at each point. The object response was measured by a small accelerometer (Isotron 35C-10) fixed at point no. 5. Data from the sensors were recorded by the Dewesoft Sirus acquisition system, with a record length of 1 s and 5 kHz frequency bandwidth.

The impact force was applied in a perpendicular direction to the composite surface while the accelerometer was attached tangentially to the vessel surface so that the measurement directions coincided with the longitudinal, radial, and circumferential directions of the cylinder, respectively.

### 2.3. Modal Analysis

In the first step, the modal shapes and corresponding frequencies were identified for vessels with a full net of measuring points (Dewesoft X3, Version: SP9). This allowed, in the next step, the correct identification of vibration modes for the remaining tanks.

Knowing the deflection pattern associated with a particular modal frequency allows us to indicate the points that were not located at the node of any considered mode. One of them, point no. 4, was chosen for further investigation.

The authors decided to choose the modal analysis method, which is based on the most widespread and useful approach, known as Circle Fit [26,27]. The circle fit method is fairly fast but becomes inadequate and inaccurate when the structure has modes that are close. Nevertheless, when the separation condition is met with the help of this method, one can determine the damping and resonance frequency based on a frequency response function (FRF). In this paper, FRFs obtained for point 4 were used to calculate the above-mentioned parameters.

## 3. Results and Discussion

The measurements for the tanks with a full mesh allowed for the identification of several modal vibration modes (modal shapes). Comparing the frequencies and corresponding motion of points on the full mesh (structures *L* 1, *H* 1) and the frequencies and motion of points on the measurement section of the remaining tanks (*L* 2, *L* 3, *H* 2, *H* 3) made it certain that the modal parameters of the same set of modes were analyzed.

**Figure 4:**
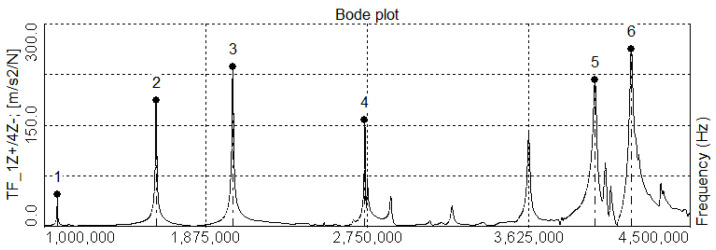
Frequency response function at point 4, vessel *L* 1.

Figure 4 shows the frequency response function determined for point 4 of vessel no. 1—winded with a low fiber tension force—3 N. The characteristic was obtained for the acceleration measured in the direction perpendicular to the composite surface. The black dots with subsequent numbers marked above indicate the frequency peaks of vibration modes selected for analysis. Based on similar curves obtained for all cylinders, it was concluded that adjacent peaks do not overlap, and the peak separation condition is met; therefore, the Circle Fit method can be used to determine the modal damping.

### 3.1. Modal Shapes

**Figure 5:**
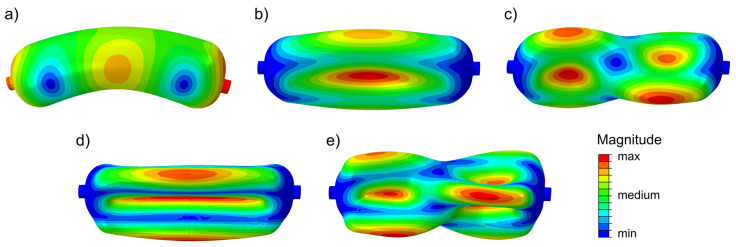
Visualization of modal selected shapes: (**a**) M 1–1 (**b**) M 2–1; (**c**) M 2–2; (**d**) M 3–1; (**e**) M 3–2.

In this research, six modal shapes were determined—in the frequency range 1–4.5 kHz (see Figure 4). For two of them, a certain general plane can be designated to which each point of the vessel surface is moving parallel. If one observes vessel motion in this plane, one can notice that points opposite the centerline of the cylinder deflect asymmetrically relative to this line. For that reason, they were called asymmetric or bending—the vessels deform like a bent beam (see Figure 5a).

For the remaining modes, more complex spatial deformation patterns can be noticed. In the case of mode M 2, the points at surfaces opposite the centerline of the cylinder are displacing symmetrically relative to this line. For the mode M 3, the asymmetric deflection of opposite vessel surfaces can be noticed (points on opposite surfaces of the cylinder are displacing asymmetrically relative to the centerline).

The visualization of the chosen vibration modes is presented in Figure 5, where contour color is related to the magnitude of movement (red—maximum, blue—minimum, for each mode separately). The numbering of modal shapes is similar to that used in the Qatu publication [28]: M 1–1, M 1–2, called here bending or asymmetric modes; M 2–1, M 2–2, M 3–1, M 3–2, called spatial modes.

### 3.2. Fundamental Frequencies

Comparing the results presented in Table 2, one can notice clear differences between the natural frequencies of the tanks with low fiber tension force (group *L*) and high tension force (group *H*).

Analysis of the results shows that the natural frequencies for first modal shapes (bending modes) are greater for vessels with elevated fiber tension (second series) than for the first series. An inverse relationship occurs in the case of spatial modes. For the *H* group, the cylinder’s natural frequencies are smaller than in the case of the first series (*L*), even though the material stiffness of the composite shell is greater for the second series (*H*).

Similar behavior was reported by Orlowska et al. [20], where prestressing causes an increase in the natural frequency associated with the first bending mode and a decrease in the natural frequencies associated with the second and third bending modes. However, Orlowska et al. investigated non-axially prestressed composite beams; therefore, a direct comparison with our case is impossible. Moreover, the frequency change mechanism in the case of tanks is not related to the initial deformation as in the case of a beam.

Qatu in his analysis of laminated composite barrel shells [28] stated that thicker shells have lower frequency parameters than thinner shells and the thickness ratio has minimal effects for the natural frequencies n = 1 (here M 1). It is consistent with the presented results, but only for asymmetric modes (M 1–1 and M 1–2). In the case of spatial modes, the results presented in this paper show an inverse relationship.

Navaneeth et al. [16] tested composite samples in cantilever boundary conditions with three values of fiber volume fraction (FVF). It was observed from the modal analysis that the vibration frequency is higher for composites with a higher FVF coefficient. However, in his case, samples with increased FVF also had bigger thickness and mass. That leads to the assumption that for bending modes, the mass reduction is not a key factor influencing natural frequencies, at least not the only one.

In our case, the composite with low fiber tension is characterized by higher mass and smaller stiffness (determined numerically and experimentally confirmed in previous work [3]) compared with group *H*; therefore, a smaller value of frequencies is assumed to be a natural consequence of both above, not the increase in mass only.

According to the paper by Nacy and Yaser [8], the stiffness of the composite plate increases due to an increase in the fiber tension during the preparation of the composite sample. A similar effect was observed by Cohen [29] in the case of composite vessels, for which the hoop modulus enlarges as a result of elevated fiber tension applied during the winding and correspond to an increase in fiber volume.

However, in this study, the amount of fiber remains constant in the case of each tested vessel (in group *L* as well as group *H*), and the fiber volume fraction coefficient changes value due to variation of the resin amount. For this reason, changes in the FVF value are not a good indicator of differences in composite stiffness.

The issue is more complicated: winding composite vessels with elevated fiber tension affects parameters such as fiber undulation angle and percentage of broken fibers, as well as changes the amount, shape, and size of defects in the composite matrix.

In the case of group *L*, the larger amount and size of voids cause weakening of the composite structure, which leads to lower stiffness and consequently lowers the natural frequencies. Yet it can also be assumed that the breaking of the fibers during the winding process with elevated fiber tension (group *H*), likewise causes a composite weakening. Both effects lead to a natural frequency shift to lower values. One can wonder which of them strongly affects the examined objects, but that aspect was not investigated.

Numerous studies have indicated that fiber prestressing improves composite architecture, such as its waviness (misalignment and undulation) and packing density, leading to strength and stiffness increase [4,6,7,8,30,31]. Therefore, one can conclude that the natural consequence mentioned above is an increase in natural frequencies in the composite vessels group *H*. However, in the case of the spatial modes, frequency shifting to lower values is visible.

According to the authors, the dominant factor influencing the change in the modal frequencies of spatial forms is the change in the composite thickness, which leads to a decrease in the bending stiffness of the composite layer. After applying increased fiber tension, the composite wall thickness decreased (see Table 1); thus, the maximum distance of tensiled fibers from the neutral axis in the bending cross-section decreased. Therefore, even though the composite material in the *H* group has a higher stiffness modulus than in the *L* group, the bending stiffness of the composite shell is lower and leads to a lower natural frequency.

In engineering applications, it may be useful to be able to control the resonant frequencies of objects such as storage tanks without having to increase mass or reduce their mechanical properties. The changes in fiber tension during the winding process result in bending modes and spatial modes, with a frequency shift in the opposite direction.

### 3.3. Modal Damping

The viscous damping (*ξ* coefficient) was determined for each considered mode of the fundamental frequency, the results obtained for each structure in groups *L* and *H* are given in Table 3.

The results of the modal analysis show, for spatial modal shapes (M 2–1 and the next ones), a visible diminishing of the damping coefficient in the case of vessels with elevated fiber tension. It is caused by a smaller matrix volume fraction in the composite shell for group *L* compared with the *H* group (Table 1). It is in agreement with Finegan and Gibson [12], as well as Timouri et al. [32] and Zhang et al. [22], who stated that the higher the volume fraction of the viscoelastic material (here, epoxy resin), the more efficient the damping is in the structure. Also, Navaneeth et al. [16] showed that for GFRP samples, the logarithmic decrement and the damping ratio decreased with increased fiber volume fraction, therefore with elevated resin fraction.

The viscoelastic nature of the matrix material is not the only cause of the difference in the damping value between groups *L* and *H*. Chandra et al. [18] sumarise recent researches and states tahat presence of cracks in matrix, debonding, breakage of fiber, delamination in composite affects the dynamic properties of the structure. Consequently, it can be assumed that elevated amounts of voids and their area, as well as lower roundness, can be another factor justifying higher damping coefficient values of vessel group *L*.

Surprisingly, the modal damping of bending forms does not show clear changes with a change in prestress level. This requires further tests and considerations.

One can notice that the ξ coefficient for M 3–1 and M 3–2 modes of the *L* 1 vessel is too low compared with the same modes of structures *L* 2 and *L* 3. The reason is not known and needs more investigation. To obtain more accurate results, the authors plan to apply one of the methods referred to as multi-degree-of-freedom curve fit in the next work.

## 4. Conclusions

This publication presents the influence of the fiber tension force change on the frequency and modal damping of the type III composite tank. Based on the experimental results, the following conclusions can be made:

- Natural frequencies of the bending modes M 1–1, M 1–2 for vessels with elevated fiber tension were greater compared with the series with low fiber tension.
- An inverse relationship occurs in the case of spatial modes M 2–1 and the next ones.
- The damping coefficient of the spatial modal shapes decreases with increasing prestress value, mainly due to changes in the matrix volume fraction in the composite shell.
- Winding the fiber with programmable tension allows one to influence not only the mass and strength of the tanks but also the modal parameters.
- The fiber tension can be used as an additional engineering parameter to shift the resonance frequencies in composite vessels.

Fiber-wound tension has a significant influence on the vessel’s modal parameters. However, further research is needed to determine precisely what mechanisms are triggered by increased tension that influence the obtained results.

## Figures and Tables

**Table 1:** Main parameters describing examined vessels.

Parameter	Group *L*	Group *H*
Fiber tension [N]	∼3	∼80
Liner thickness [mm]	∼2.02	∼2.05
Cylinder inner diameter [mm]	100	100
Cylinder length [mm]	500	500
Winding angle [°]	54	54
Number of composite layers [-]	7	7
Curing temperature	30 °C	30 °C
Composite thickness [mm]	∼4.75	∼3.51
Fiber volume fraction [%]	∼45	∼63
Epoxy resin	EPIKOTE Resin L 20	EPIKOTE Resin L 20
Carbon fiber	Torayca 700S 24 k	Torayca 700S 24 k
Material of liner	steel	steel

**Table 2:** Natural frequencies identified for vessels with *L*—low and *H*—high fiber tension.

	Frequency [Hz] of Mode:					
Vessel	M 1–1	M 1–2	M 2–1	M 2–2	M 3–1	M 3–2
*L* 1	1069.6	2737.4	1604.8	2020.5	3983.5	4187.6
*L* 2	1073.5	2727.2	1566.6	1995.8	3918.2	4127.6
*L* 3	1060.5	2735.6	1579.1	2001.6	3940.3	4140.5
*H* 1	1089.4	2796.1	1406.9	1866.7	3582.6	3782.0
*H* 2	1108.2	2802.0	1405.8	1874.7	3582.1	3788.8
*H* 3	1098.6	2809.6	1388.7	1868.0	3551.4	3760.1

**Table 3:** Modal damping determined for vessels with *L*—low and *H*—high fiber tension.

	Viscous Damping *ξ* [–] of Mode:					
Vessel	M 1–1	M 1–2	M 2–1	M 2–2	M 3–1	M 3–2
*L* 1	$1.05\times 10^{-3}$	$7.76\times 10^{-4}$	$1.82\times 10^{-3}$	$1.61\times 10^{-3}$	$2.47\times 10^{-4}$	$2.22\times 10^{-4}$
*L* 2	$1.15\times 10^{-3}$	$1.05\times 10^{-3}$	$1.81\times 10^{-3}$	$1.81\times 10^{-3}$	$4.46\times 10^{-3}$	$2.82\times 10^{-3}$
*L* 3	$1.33\times 10^{-3}$	$8.12\times 10^{-4}$	$1.75\times 10^{-3}$	$1.91\times 10^{-3}$	$2.53\times 10^{-3}$	$2.45\times 10^{-3}$
*H* 1	$9.82\times 10^{-4}$	$9.66\times 10^{-4}$	$1.15\times 10^{-3}$	$1.21\times 10^{-3}$	$1.65\times 10^{-3}$	$1.40\times 10^{-3}$
*H* 2	$1.33\times 10^{-3}$	$1.02\times 10^{-3}$	$1.22\times 10^{-3}$	$1.29\times 10^{-3}$	$1.62\times 10^{-3}$	$1.78\times 10^{-3}$
*H* 3	$1.36\times 10^{-3}$	$6.77\times 10^{-4}$	$1.10\times 10^{-3}$	$1.17\times 10^{-3}$	$1.61\times 10^{-3}$	$1.61\times 10^{-3}$

## Data Availability

Data is available within the article.
